# Sedative medications: a potentially modifiable risk factor for improving neurocognitive outcomes after critical illness in infants and children

**DOI:** 10.3389/fped.2026.1874293

**Published:** 2026-07-20

**Authors:** Alana GaHyun Byeon, Marina Mir, Angela Jerath, Marat Slessarev, Saptharishi Lalgudi Ganesan, Nicole K. McKinnon

**Affiliations:** 1 Department of Critical Care Medicine, Hospital for Sick Children, Toronto, ON, Canada; 2 Division of Pediatric Critical Care, McMaster Medical Center, Hamilton, ON, Canada; 3 Schulich Heart Program, Sunnybrook Research Institute, Sunnybrook Health Sciences Centre, Toronto, ON, Canada; 4 Department of Anesthesiology and Pain Medicine, Temerty Faculty of Medicine, University of Toronto, Toronto, ON, Canada; 5 ICES, Sunnybrook Health Sciences Centre, Toronto, ON, Canada; 6 Department of Critical Care Medicine, Schulich School of Medicine and Dentistry, Western University, London, ON, Canada; 7 Department of Paediatrics & Dept. of Clinical Neurological Sciences, Schulich School of Medicine and Dentistry, Western University, London, ON, Canada; 8 Division of Child Health & Therapeutics, Children’s Health Research Institute, London Health Sciences Centre Research Institute, London, ON, Canada; 9 Department of Paediatrics, and Department of Physiology, Temerty Faculty of Medicine, University of Toronto, Toronto, ON, Canada; 10 Department of Neuroscience and Mental Health, Sickkids Research Institute, Toronto, ON, Canada

**Keywords:** anesthetics, critical care, delirium, neurocognitive outcomes, pediatrics, sedatives

## Abstract

Critically ill infants and children often need prolonged sedation to support life-saving therapeutic interventions. Although advances in pediatric intensive care (PICU) have substantially improved survival, an increasing body of evidence indicates that many survivors experience persistent neurocognitive and neurobehavioral impairments. These outcomes arise from a complex interplay between pre-morbid status, critical illness related physiological stressors and iatrogenic exposures during periods of brain development. Evidence consistently links benzodiazepines to increased risk of delirium, sleep fragmentation, withdrawal, and adverse cognitive trajectories in pediatric populations. In contrast, *α*_2_-adrenergic agonists such as dexmedetomidine may be associated with a lower risk of delirium, improved sleep architecture, and potential neuroprotective effects in preclinical and early clinical studies, although human evidence is conflicted with limited data in PICU. Although several studies have evaluated the long-term neurocognitive effects of inhaled anesthetics after general surgical anesthesia, their increasing use for PICU sedation remains understudied. This narrative review synthesizes preclinical and clinical literature examining the associations between sedation practices in PICUs and subsequent neurocognitive outcomes. We focus on potential modifiable contributors, including sedative class selection, sedative polypharmacy, sleep disruption, and delirium, while acknowledging non-modifiable risk factors such as developmental stage at illness onset, acute neurological injury, systemic inflammation, and non-clinical social determinants of health.

## 1 Introduction

Critically ill infants and children routinely receive prolonged sedation, over days to weeks, often involving multiple agents, to support life-saving care. While brief exposures are considered low risk, prolonged exposure places patients at heightened risk for delirium, iatrogenic withdrawal, and long-term neurocognitive impairment (1). Deficits in memory, executive function, attention, and emotional regulation have been documented in a significant proportion of pediatric intensive care unit (PICU) survivors, including among those without pre-existing neurological conditions (2, 3). Importantly, infants and children with underlying vulnerabilities may be at even higher risk for further neurocognitive decline. The developing brain is particularly susceptible to both pathophysiological disturbances like hypoxia, hypotension, acidosis, systemic inflammation and iatrogenic exposures (4–6). Modifiable contributors, including sedation-associated delirium, sleep fragmentation, and environmental stressors, further complicate the neurodevelopmental trajectory of pediatric survivors of critical illness.

Although research supports associations between PICU admission and neurocognitive deficits, methodological variability and limited mechanistic insights hinder direct translation into bedside clinical practice (7). Preclinical studies suggest sedative agents can disrupt brain development, but translating these findings to critically ill children is complex. Clinical data are often confounded by illness severity, comorbidities, patient age, and care heterogeneity. Clinical trials of inhalational sedatives, which are increasingly more commonly used in critical care, have focused only on short exposures in otherwise healthy children.

This narrative review builds on evolving preclinical and clinical research on neurocognitive outcomes following critical illness and sedation in the PICU, highlighting both modifiable and non-modifiable risk factors, with an emphasis on modifiable factors that can inform bedside practice.

For this review, we employed an iterative search strategy, beginning with a broad examination of the literature on neurocognitive outcomes following adult and pediatric critical illness and post-intensive care syndrome. We used literature identified through successive rounds of database querying (PubMed) and reference tracing rather than a single predefined protocol. Priority was given to manuscripts reporting randomized controlled trials and large cohort studies, particularly those that have been highly cited or incorporated into clinical practice guidelines, to anchor the review in the most influential and methodologically robust clinical evidence. Where a mechanistic context was needed to link clinical outcomes to underlying neurodevelopmental processes, relevant preclinical studies were also included. Articles were selected based on their relevance to the review's central themes, with an emphasis on synthesizing evidence across clinical and preclinical domains.

## 2 Sedation-related modifiable risk factors

### 2.1 Intravenous and oral sedatives

Sedative choice in the PICU has historically been dominated by midazolam infusions (8). Benzodiazepines are increasingly recognized as contributors to adverse cognitive outcomes, including lower IQ scores in children 3–8 years after being treated with a sedation strategy that included only opioids and benzodiazepines during their PICU admission, compared with children who were treated with multiple sedative classes (9).

Benzodiazepine exposure is independently associated with increased incidence of pediatric sedation-associated delirium (pSAD) with incidence rates exceeding 50% in ventilated children (10–12). Specifically, Mody et al. (10) reported that each log increase in benzodiazepine dosage was associated with a 43% higher risk of delirium (10). Drury et al. (5) found that higher cumulative benzodiazepine exposure was independently associated with worse acute-phase cognitive outcomes (5), while Long et al. (13) found an increased risk for post-traumatic stress syndrome in PICU survivors who were exposed to midazolam, one-month post-discharge (13). Based on these concerns, dexmedetomidine, an alpha-2 adrenergic agonist, has been recommended as the first-line agent for sedation in critically ill children and for light sedation or to decrease delirium risk in adults requiring mechanical ventilation in the Society for Critical Care Medicine Clinical Practice Guidelines (14, 15).

Alpha-2 adrenergic agonists, including dexmedetomidine and clonidine, have been suggested to have a lower risk of delirium and potential neuroprotective effects (16–18). In neonatal rodent and hypoxic-ischemic encephalopathy (HIE) animal models, dexmedetomidine administration has been shown to reduce apoptosis and oxidative stress, decrease lesion size, and improve functional recovery (19–21). Mechanistically, dexmedetomidine increases anti-apoptotic proteins, reduces p53-mediated cell death pathways, limits glutamate excitotoxicity, and inhibits calcium influx via NMDA receptors (22). These pathways directly target processes implicated in delirium pathophysiology, including neuronal hyperexcitability and neuroinflammation.

While pre-clinical data show clear benefits in terms of apoptosis, oxidative stress and neuroinflammation compared to other sedative agents, including inhalational sedatives, the clinical data remain mixed. In adults, dexmedetomidine demonstrated superiority over lorazepam in achieving sedation targets, reducing delirium incidence, and showing trends toward decreased mortality in the MENDS trial (23). In the recently published A2B trial, neither dexmedetomidine nor clonidine was superior to propofol in reducing time to successful extubation, and both were associated with higher rates of patient agitation, with no observed mortality benefit (24). Studies in pediatric patients are limited. The largest study of dexmedetomidine in critically ill children, the PROSDEX study, included 163 patients, with a median age of 13 months (interquartile range 4- 71 months) across 9 PICUs in Italy. The prospective observational study included a heterogeneous patient population, with 62% invasively mechanically ventilated, 34% supported with non-invasive mechanical ventilation and 18% with high flow nasal cannula. The study reported that patients who received at least 24 h of dexmedetomidine required less benzodiazepines, opioids, propofol and ketamine, in addition to having lower rates of withdrawal and delirium as compared to the 24 h prior to the initiation of dexmedetomidine (25). Several small clinical trials have found similar results (26–29), however, a recent meta-analysis showed that, with pooled analysis, dexmedetomidine had little effect on duration of mechanical ventilation, that it may reduce the risk of delirium, and the impact on withdrawal is uncertain. Additionally, this meta-analysis highlighted that the certainty of the evidence was often low or very low (30). Further large randomized controlled trials with standardized titration protocols are required to fully understand whether the benefits shown in preclinical studies extend to the bedside. Currently, the Dexmedetomidine Use in Infants Undergoing Cooling Due to Neonatal Encephalopathy (DICE) trial is recruiting; however, these results will have limited applicability to patients admitted to the PICU (31).

### 2.2 Inhaled anesthetics

Inhaled anesthetics such as isoflurane and sevoflurane remain widely used for surgical anesthesia but are being increasingly utilized in critical illness for sedation via miniature vaporizing devices such as the SedaConDa® (32). Inhaled agents exert broad central nervous system effects by enhancing inhibitory neurotransmission via gamma-aminobutyric acid (GABA) and glycine receptors, while simultaneously suppressing excitatory signaling through blockade of N-methyl-D-aspartate (NMDA), nicotinic acetylcholine, glutamate, and serotonin (5-HT_3_) receptors (21). While these pharmacologic mechanisms are advantageous for inducing anesthesia, they have also been implicated in neurotoxicity, particularly in the developing brain. From preclinical studies of brain anatomy following inhaled anesthetic exposure to large, multicenter clinical trials, significant research has sought to understand and quantify this risk. Despite this, no clear answer exists.

Preclinical studies of inhaled anesthetic exposure in neonatal rodent models suggest disruptions in synaptic plasticity and activation of neuroapoptotic pathways during key periods of synaptogenesis (33–35). The findings, however, caution against generalization to children, as these models often employ inhaled anesthetic exposures that significantly exceed typical clinical doses (>3 minimum alveolar concentration, MAC) and do not reflect human neurodevelopmental timelines (36).

Large-scale clinical trials assessing brief exposures (less than 240 min) in healthy children have been largely reassuring. The neurodevelopmental outcome at 5 years of age after general anesthesia or awake-regional anesthesia in infancy (GAS trial) demonstrated no significant difference in intelligence quotient (IQ) scores at 5 years of age between infants who underwent general anesthesia with sevoflurane and those who received regional anesthesia during inguinal hernia repair (37, 38). Similarly, the Pediatric Anesthesia Neurodevelopment Assessment (PANDA) study, a sibling-matched cohort analysis, found no statistically significant difference in IQ scores at 10 years of age between children exposed to a single general anesthetic under 3 years of age and their unexposed siblings (39). The Mayo Anesthesia Safety in Kids (MASK) study extended these findings, observing no substantial IQ deficits, 5–17 years after either a single or multiple brief exposures, but identifying potential domain-specific vulnerabilities, such as processing speed and fine motor skills (40). Recently, two trials have compared neurocognitive outcomes after a combination of sevoflurane, dexmedetomidine, and remifentanil vs. sevoflurane alone to determine whether limiting exposure to inhalational anesthetic agents affects neurocognitive outcomes (41, 42). The Trial Remifentanil DEXmedetomidine trial (TREX) to date has reported only short-term outcomes related to intraoperative hypotension and bradycardia, finding that patients exposed to sevoflurane alone had a higher incidence of intraoperative hypotension but less bradycardia than those exposed to sevoflurane, dexmedetomidine, and remifentanil (42). Sang-Hwan and colleagues reported no differences in neurodevelopmental status between children exposed to sevoflurane, dexmedetomidine, and remifentanil (DEX-R) before 24 months of age, vs. those exposed to sevoflurane alone, on the Korean Leiter International Performance Scale and the Child Behaviour Checklist at 28–30 months of age (41). Neither trial has yet to report its primary outcome, which is full-scale IQ testing at 3 years of age (TREX) and 5 years of age (Sang-Hwan et al.). Importantly, all these trials excluded children with significant comorbidities, organ dysfunction or critical illness, limiting their generalizability to the PICU population. Additionally, the inhalational anesthetic exposures during these trials were short (averaging around 50 min), whereas sedation during critical illness is continuous, involves multiple agents, and may be required for prolonged periods (days- weeks). A meta-analysis of 31 studies of neurodevelopment following inhalational anesthetic exposure suggested a higher risk of neurodevelopmental disorders, learning problems and ADHD in children who experience prolonged or repeated anesthesia (43). Unfortunately, neurodevelopmental disorders are also associated with socioeconomic status and other PICU and home environmental factors (44, 45), and these confounders are rarely addressed in studies of anesthetic exposure.

Clinical concerns about prolonged inhaled sedation are supported by observational studies in neonates post-cardiac surgery, where cumulative inhaled anesthetic exposure has been associated with MRI-detected white matter changes and poorer neurodevelopmental outcomes at one year (46, 47). Furthermore, rare complications of inhaled anesthetic use in intensive care unit for sedation include emergence delirium, hallucinations, and psychomotor dysfunction including choreoathetois, although robust pediatric data regarding types and incidence of such complications remain extremely limited (21, 48, 49).

Two recent randomized controlled trials (RCT) have attempted to better understand the benefits and risks of the use of inhaled agents for ongoing sedation in critically ill patients. The SESAR trial, a multicenter randomized controlled study of adults with moderate-to-severe acute respiratory distress syndrome (ARDS), found that patients sedated with inhaled sevoflurane had fewer ventilator-free days at 28 days and lower 90-day survival compared to those receiving intravenous propofol (50). The generalizability of this study is severely limited by key methodological factors: over half the cohort had COVID-19 pneumonia, and the protocol mandated deep sedation (RASS −4 to −5) with neuromuscular blockade for 48 h regardless of clinical need. This sedation approach diverges from current sedation best practices in both adults and children, which recommend protocolized sedation targeted to validated sedation scores, including the Richmond Agitation-Sedation Scale (RASS), State Behavioral Scale (SBS), or COMFORT-B scale, with a focus on strategies to minimize overall sedation and reduce coma whenever clinically feasible (14, 15).

In children, the ISOComfort trial offers the first randomized evidence on extended use of inhaled sedation in mechanically ventilated children for up to 54 h. Isoflurane was found to be non-inferior to midazolam in efficacy and safety (51). However, the trial excluded infants and toddlers, did not assess long-term neurodevelopmental outcomes, and used benzodiazepines as the comparator, a medication class which has been linked to delirium in children (10, 11) and is not routinely recommended for sedation in critically ill children (15). The Advancing Brain Outcomes in pediatric critically ill patients sedated with Volatile AnEsthetic Agents (ABOVE) trial is a pilot, feasibility trial (NCT05867472), that assesses the feasibility of enrolling children in an RCT that will compare neurocognitive outcomes in children sedated with isoflurane to standard sedative practice (15). In contrast to ISOComfort, it is anticipated that a portion of participants randomized to standard of care will be sedated with dexmedetomidine in accordance with current guidelines (15). Additional exploratory endpoints in the pilot feasibility trial include neurocognitive outcomes at 1 year after discharge, making it the first trial in critically ill children to evaluate neurocognition following prolonged isoflurane exposure (Table 1).

**Table 1:** Clinical trials of inhaled anesthetic agents in children.

Trial	Study Design	Population	N	Setting	Intervention vs. Control	Duration of Exposure	Primary Outcome	Age at Assessment	Key Findings	Status
GAS	Multicenter RCT	Infants ≤60 weeks PMA	722	OR	Sevoflurane GA vs. Awake-regional anesthesia	Median 54 min (IQR 41–70)	Full-Scale IQ (WPPSI-III)	5 years	No difference in FSIQ: sevoflurane 98.87 vs. regional 99.08 (difference 0.23, 95% CI −2.59 to 3.06). Equivalence demonstrated within ±5-point margin.	Complete
PANDA	Ambidirectional sibling-matched cohort	Single GA exposure before age 36 months	105 sibling pairs	OR	Single inhaled GA vs. Unexposed sibling	Median 80 min (range 20–240)	Full-Scale IQ (WISC-IV/WASI)	8–15 years	No difference in FSIQ: exposed 111.0 vs. unexposed 110.8 (difference −0.2, 95% CI −2.6 to 2.9, *P* = 0.83). No differences in neurocognitive or behavioral secondary outcomes.	Complete
MASK	Propensity-matched retrospective cohort	Inhaled GA exposure before age 3 years	997	OR	Single or multiple inhaled GA exposures vs. Unexposed controls	Median cumulative duration 45–187 min	Weschler Abbreviated Scale of intelligence (IQ composite)	8–12 or 15–20 years	No difference in IQ across groups. Multiple (not single) exposures associated with decreased processing speed and fine motor coordination. Parents of multiply-exposed children reported more learning/behavioral difficulties.	Complete
TREX	Multicenter RCT (single-blinded)	Children <2 years requiring ≥2 h of GA	455	OR	Low-dose sevoflurane + dexmedetomidine + remifentanil (LD-SEVO) vs. sevoflurane	Median 2.8 h	Full-Scale IQ (WPPSI-IV at years)	3 years (pending)	Perioperative outcomes broadly similar between groups. LD-SEVO had lower MAC-hour exposure. Primary neurodevelopmental outcome (FSIQ at age 3) pending.	*Ongoing*
DEX-R	Single-center RCT	Children <2 years requiring GA	400	OR	Sevoflurane + dexmedetomidine + remifentanil vs. sevoflurane	Mean 73–77 min	Full-Scale IQ (WPPSI-IV at 5 years)	28–30 months (secondary); 5 years (primary, pending)	Secondary outcomes at 28–30 months: no significant difference in IQ (103.6 vs. 102.5, *P* = 0.442) or Bayley-III scores. Primary outcome (FSIQ at age 5) pending.	*Ongoing*
ISOComfort	Multicenter, open-label RCT	Mechanically ventilated PICU patients (3–17 years)	92	PICU	Inhaled isoflurane vs. IV midazolam	Up to 54 h	Adequate sedation (COMFORT-B ≤ 23 in ≥80% assessments)	During PICU stay (no long-term follow-up)	Isoflurane noninferior to midazolam for sedation adequacy (77.6% vs. 58.3%). Shorter ventilation, faster PICU discharge. No long-term neurocognitive outcomes assessed.	Complete
ABOVE	Multicenter pilot RCT	Mechanically ventilated PICU patients <18 years	Target: 60	PICU	Inhaled isoflurane vs. IV sedation	—	Feasibility and recruitment; neurocognition at 1 year (secondary)	1 year (secondary)	Currently recruiting. Pilot study to assess feasibility of a larger trial evaluating neurocognitive outcomes of inhaled volatile sedation in critically ill children.	*Recruiting*

GA, general anesthesia; FSIQ, Full-Scale Intelligence Quotient; IQR, interquartile range; MAC, minimum alveolar concentration; OR, operating room; PICU, pediatric intensive care unit; PMA, postmenstrual age; WPPSI, Wechsler Preschool and Primary Scale of Intelligence; WISC, Wechsler Intelligence Scale for Children; WASI, Wechsler Abbreviated Scale of Intelligence; COMFORT-B, COMFORT Behavior scale.

### 2.3 Sedative polypharmacy

The simultaneous use of multiple sedative and analgesic classes, sedative polypharmacy, is common in PICUs and represents a potential modifiable contributor to neurocognitive risk. In a study of 1,300 critically ill children, Bose et al. (52) found that exposure to ≥4 sedative classes was associated with a 72.6% incidence of delirium (52). Notably, polypharmacy mediated nearly 40% of the delirium risk attributed to mechanical ventilation, emphasizing its central role in neurocognitive vulnerability. Additionally, more than 75% of PICU patients encounter potentially harmful drug–drug interactions (53), complicating efforts to isolate specific neurotoxic effects.

### 2.4 Sleep disruption and fragmentation

**Figure 1:**
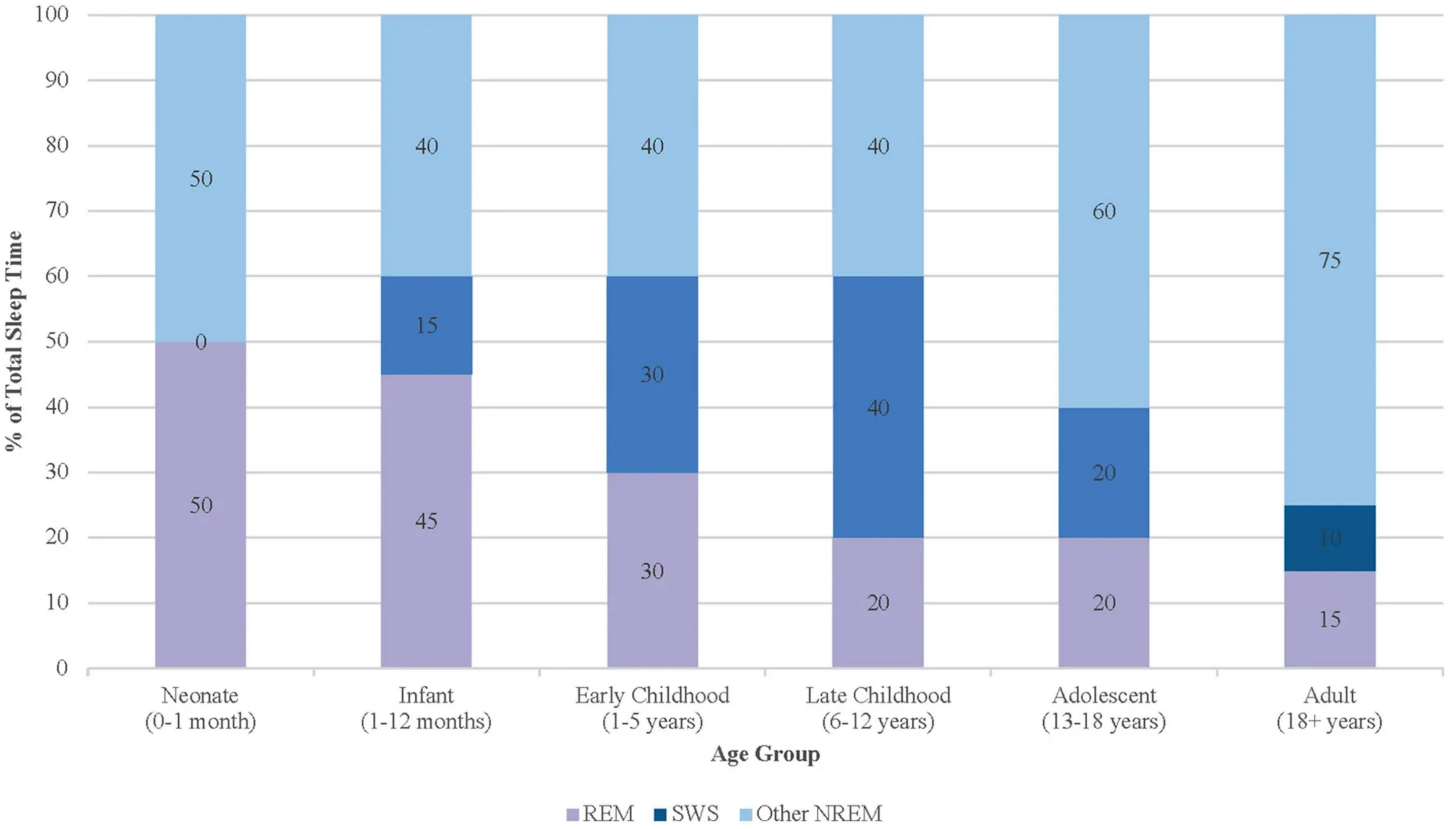
Age-related changes in sleep stage distribution from infancy through adulthood, with peaks in SWS during late childhood (60).

Sleep is an underexplored determinant of outcomes in critically ill children, where restorative sleep is essential for brain maturation. The development of circadian rhythms and sleep regulation begins *in utero* and undergoes marked changes across childhood to support brain maturation and physical growth (54). Sleep architecture is shaped by a combination of neurological pathways, hormonal influences, and behavioral routines, but these patterns evolve with age to meet changing developmental needs (55). Light sleep, or non-rapid eye movement sleep stage 1 and 2 (NREM1 and 2) is marked by sleep spindles and K-complexes that consolidate by 3 months of age, reflecting increasing sensitivity to environmental disruption. Deep sleep, or slow wave sleep (SWS)/NREM 3) is characterized by high-amplitude delta waves (0.5–4 Hz) and predominates in early childhood, when it is most critical for growth, immune regulation, and synaptic pruning. By adolescence, SWS declines by 40% as cortical remodeling occurs. Notably, children spend proportionally more time in rapid eye movement (REM) sleep during infancy and more SWS during early childhood (Figure 1) (56–59).

In the PICU, sleep fragmentation, characterized by frequent awakenings, reduced REM, and circadian disruption, has been associated with neuroinflammation and impaired memory consolidation (60–63). Children may be uniquely vulnerable, as disrupted sleep during critical periods of synaptic plasticity can alter long-term cognitive trajectories (64). Environmental and treatment-related factors further compound this risk: constant light, loud noise, and repeated interventions fragment sleep, while mechanical ventilation is strongly linked to delirium and prolonged PICU stay (3, 60).

Moreover, sedation is often diametrically opposite of physiologic sleep from a neurophysiological standpoint; commonly used agents suppress REM and SWS stages, producing EEG patterns distinct from restorative sleep and potentially impairing neurodevelopment (65). Sedative and analgesic agents exert profound effects on sleep architecture. Benzodiazepines induce beta frequency activity on EEG, reduce REM and increase lighter NREM 2 sleep; opioids further suppress SWS and REM; and propofol produces diffuse slow-wave EEG activity with loss of REM and NREM 1 stages (60). Greater sedative exposure is consistently associated with worsening fragmentation, prolonged ventilation, and agitation, while polypharmacy compounds these effects. In contrast, *α*₂-agonists such as dexmedetomidine preserve NREM 2 sleep and approximate a more physiologic sleep profile in children (65), suggesting a potential protective role.

EEG-based anesthetic depth monitoring, which is increasingly used to improve sedation during general anesthesia, offers little insight into sleep quality. Devices such as BIS, SedLine, and Narcotrend condense EEG activity into a single index to prevent deeper anesthesia, thereby leading to burst suppression and lighter anesthesia, which can manifest as intraoperative awareness. These EEG-based monitors have several limitations regarding their utility in monitoring sedation depth and sleep in critically ill infants and children. The devices have been developed and validated for adult-based brain algorithms and therefore have limited utility in infants and younger children whose EEG amplitudes and frequencies differ during critical periods of brain maturation (66). Additionally, EEG-based monitors focus on sedative-induced unconsciousness, as synchronized slow-wave patterns that lack physiologic sleep features such as spindles or K-complexes (67). Therefore, sophisticated, pediatric-specific, real-time EEG monitoring tools that can not only quantify anesthetic depth but also objectively evaluate sleep quality at the bedside are urgently needed (68). Intervention strategies targeting sleep, including minimizing nighttime disturbances, simulating light–dark cycles, and implementing pediatric liberation bundles, also represent emerging opportunities to mitigate sleep disruption and improve sleep quality in critically ill patients. By addressing both environmental and pharmacologic contributors, such approaches may protect against long-term neurocognitive impairment in PICU survivors.

### 2.5 Delirium

Delirium is a frequent and serious complication of pediatric critical illness, defined as acute cerebral dysfunction with disturbances in attention, awareness, and consciousness. In both pediatric and adult survivors of critical illness, delirium strongly predicts long-term cognitive impairment and reduced quality of life (69–71). The pathophysiology of delirium increasingly points to inflammatory and neuronal injury pathways. Elevated levels of IL-6, IL-8, MCP-1, TNF-α, cortisol, and S100-*β* have been observed in critically ill children with delirium (4, 72). These mediators disrupt the blood–brain barrier, activate glial cells, and promote neuroinflammation. Systemic inflammation may also amplify the neurocognitive risks of sedative and analgesic exposure (73).

Sedative medications are among the strongest modifiable contributors to delirium in the PICU (10, 74). Large cohort studies demonstrate that delirium occurs in up to 23% of mechanically ventilated patients, with benzodiazepine exposure, mechanical ventilation, and illness severity (PRISM III scores) independently associated with risk (3). Benzodiazepines show a clear dose–response effect with each incremental daily increase raising delirium risk by 43%, and overall odds nearly fivefold higher compared to unexposed children (10, 12). Children who developed delirium had longer PICU stays, extended ventilation, and increased mortality. Importantly, benzodiazepine exposure independently predicted worse executive function in PICU survivors, even after adjusting for confounders (5). The ongoing movement toward benzodiazepine-sparing sedation highlights recognition of these risks. Protocols favoring *α*₂-adrenergic agonists such as dexmedetomidine may reduce the risk of delirium (30), but given the complex interplay of environmental and physiologic factors that cause delirium, switches in sedative regimens alone will not eliminate this PICU morbidity.

### 2.6 Socioeconomic and environmental determinants

Non-biological factors, including socioeconomic status and environmental exposures, play a substantial role in shaping recovery. Lower socioeconomic status has been independently linked to worse neurocognitive outcomes, even after adjustment for illness severity (2). Contributing mechanisms include reduced access to post-discharge rehabilitation, inadequate educational support, and healthcare disparities. Environmental exposures, including early-life stress, parental education levels, and housing instability, further compound these risks and may synergistically interact with biological vulnerabilities established during critical illness (75).

## 3 Non-modifiable factors

### 3.1 Acute neurological injuries

Acute neurological insults acquired during or preceding critical illness including traumatic brain injury, ischemic or hemorrhagic stroke, hypoxic-ischemic events, and central nervous system infections directly impact neuronal integrity and network development. These conditions are associated with persistent impairments in memory, attention, processing speed, and executive functioning (47). In a cohort study by Williams et al. (76), approximately 35% of children admitted to the PICU with primary neurologic diagnoses developed new disabilities by hospital discharge, with the highest rates observed in infectious, inflammatory, or cerebrovascular conditions (76).

### 3.2 Developmental stage at illness onset

The timing of critical illness relative to neurodevelopmental milestones significantly influences long-term cognitive outcomes. Infants and young children, particularly under three years of age are in a phase of rapid brain development involving synaptogenesis, myelination, and cortical network formation (6). Disruptions during this critical window can lead to permanent alterations in brain architecture and function. Dervan and colleagues demonstrated that younger age was independently associated with both increased risk of PICU delirium and poorer neurocognitive outcomes post-discharge (3). Experimental studies further support these findings, demonstrating greater susceptibility to anesthetic and inflammatory insults when exposures occur during critical periods of neurodevelopment (21). Although plasticity may support partial recovery, the initial developmental insult often determines the trajectory of functional outcomes.

### 3.3 Critical illness

Critical illness is characterized by recurrent episodes of hypoxia, hypotension, and metabolic derangements, all of which can precipitate global and focal cerebral dysfunction and even ischemia. These physiological insults compromise cerebral autoregulation, reduce oxygen delivery, and disrupt neuronal metabolism, thereby contributing to structural brain injury and impaired cognitive development (77). Choong et al. (78) found that the severity, timing, and duration of hypoxic or hypotensive episodes strongly predicted neurodevelopmental outcomes among PICU survivors (78).

Furthermore, systemic inflammation during critical illness can disrupt the blood-brain barrier facilitating peripheral immune cell infiltration and activating resident microglia and astrocytes. This neuroinflammatory cascade exacerbates neuronal apoptosis, impairs synaptic connectivity, and may induce long-term changes in neural circuits responsible for cognition and behavior (4).

## 4 Conclusion

**Figure 2:**
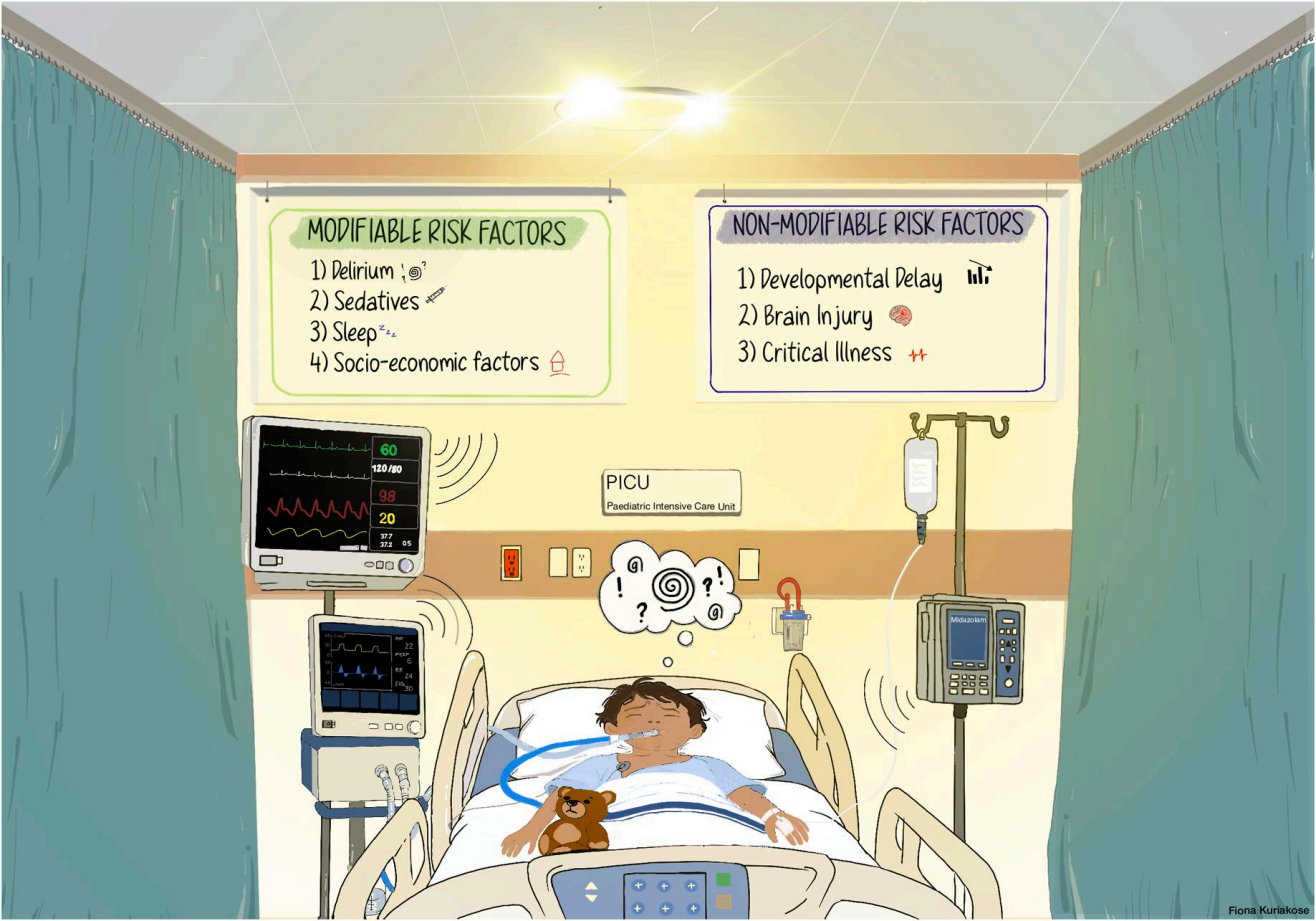
Modifiable and non-modifiable risk factors for improving neurologic outcomes in children after critical illness. The young patient, who is mechanically ventilated and sedated with midazolam, is delirious and experiencing sleep fragmentation from the bright lights and alarms on the ventilator, monitor and IV pumps. While developmental stage at the time of illness, acute brain injury, hypoxia, and hypotension are difficult to modify, using a benzodiazepine-sparing sedation strategy, reducing excessive alarms, focusing on sleep hygiene, and screening for socio-economic factors and home supports may improve long-term cognitive outcomes.

As survival from pediatric critical illness continues to improve, attention must shift decisively toward protecting the developing brain of survivors. The evidence reviewed here underscores that neurocognitive morbidity arises not from a single exposure but from the cumulative interaction of illness-related physiological stressors with iatrogenic factors such as sedative choice, polypharmacy, sleep disruption, and delirium. While some risks such as age at illness, acute neurological injury, and severity of hypoxia or inflammation are essentially non-modifiable, many others are amenable to intervention. Benzodiazepine-sparing sedation strategies, preference for agents that better preserve sleep and reduce delirium, minimization of sedative polypharmacy, and environmental and organizational efforts to protect circadian rhythm and restorative sleep represent actionable opportunities to reduce long-term harm (Figure 2). Moving forward, progress will require integrating neurodevelopmental outcomes into sedation trials, extending follow-up beyond hospital discharge, and leveraging bedside tools such as EEG-informed monitoring to titrate sedation and promote sleep. A developmentally informed, liberation-focused approach to pediatric sedation grounded in delirium prevention and sleep preservation offers a realistic pathway to improving not only survival but also the cognitive and functional outcomes of infants and children who require critical care.

## References

[1] Needham DM Davidson J Cohen H Hopkins RO Weinert C Wunsch H. Improving long-term outcomes after discharge from intensive care unit. Crit Care Med. (2012) 40(2):502–9. 10.1097/CCM.0b013e318232da75 21946660

[2] Madurski C Treble-Barna A Fink EL. Cognitive impairment following pediatric critical illness. Pediatr Crit Care Med. (2018) 19(3):277–8. 10.1097/PCC.0000000000001436 29499029 PMC5836494

[3] Dervan LA Gennaro JLD Farris RWD Watson RS. Delirium in a tertiary PICU: risk factors and outcomes*. Pediatr Crit Care Me. (2020) 21(1):21–32. 10.1097/PCC.0000000000002126 31568239

[4] Brummel NE Hughes CG McNeil JB Pandharipande PP Thompson JL Orun OM. Systemic inflammation and delirium during critical illness. Intensiv Care Med. (2024) 50(5):687–96. 10.1007/s00134-024-07388-6 PMC12416785 38647548

[5] Drury KM Hall TA Orwoll B Adhikary S Kirby A Williams CN. Exposure to sedation and analgesia medications: short-term cognitive outcomes in pediatric critical care survivors with acquired brain injury. J Intensiv Care Med. (2024) 39(4):374–86. 10.1177/08850666231210261 PMC11132562 37885235

[6] Volpe JJ. Dysmaturation of premature brain: importance, cellular mechanisms, and potential interventions. Pediatr Neurol. (2019) 95:42–66. 10.1016/j.pediatrneurol.2019.02.016 30975474

[7] Royer AS-M Busari JO. A systematic review of the impact of intensive care admissions on post discharge cognition in children. Eur J Pediatr. (2021) 180(12):3443–54. 10.1007/s00431-021-04145-5 34114079 PMC8192269

[8] Kudchadkar SR Yaster M Punjabi NM. Sedation, sleep promotion, and delirium screening practices in the care of mechanically ventilated children. Crit Care Med. (2014) 42(7):1592–600. 10.1097/CCM.0000000000000326 24717461 PMC4061156

[9] Curley MAQ Beers SR Asaro LA Burns C Koh MJ Angus DC. Sedative choice and neurocognitive outcomes after critical illness in early childhood. JAMA Netw Open. (2026) 9(5):e2613599. 10.1001/jamanetworkopen.2026.13599 42154465 PMC13187875

[10] Mody K Kaur S Mauer EA Gerber LM Greenwald BM Silver G. Benzodiazepines and development of delirium in critically ill children. Crit Care Med. (2018) 46(9):1486–91. 10.1097/CCM.0000000000003194 29727363 PMC6095819

[11] Traube C Silver G Gerber LM Kaur S Mauer EA Kerson A. Delirium and mortality in critically ill children. Crit Care Med. (2017) 45(5):891–8. 10.1097/CCM.0000000000002324 28288026 PMC5392157

[12] Traube C Silver G Reeder RW Doyle H Hegel E Wolfe HA. Delirium in critically ill children. Crit Care Med. (2017) 45(4):584–90. 10.1097/CCM.0000000000002250 28079605 PMC5350030

[13] Long D Gibbons K Brocque RL Schults JA Kenardy J Dow B. Midazolam exposure in the paediatric intensive care unit predicts acute post-traumatic stress symptoms in children. Aust Crit Care. (2022) 35(4):408–14. 10.1016/j.aucc.2021.06.004 34373171

[14] Lewis K Balas MC Stollings JL McNett M Girard TD Chanques G. A focused update to the clinical practice guidelines for the prevention and management of pain, anxiety, agitation/sedation, delirium, immobility, and sleep disruption in adult patients in the ICU. Crit Care Med. (2025) 53(3):e711–27. 10.1097/CCM.0000000000006574 39982143

[15] Smith HAB Besunder JB Betters KA Johnson PN Srinivasan V Stormorken A. 2022 Society of critical care medicine clinical practice guidelines on prevention and management of pain, agitation, neuromuscular blockade, and delirium in critically ill pediatric patients with consideration of the ICU environment and early mobility. Pediatr Crit Care Me. (2022) 23(2):e74–110. 10.1097/PCC.0000000000002873 35119438

[16] Chen Y Zhang X Zhang B He G Zhou L Xie Y. Dexmedetomidine reduces the neuronal apoptosis related to cardiopulmonary bypass by inhibiting activation of the JAK2–STAT3 pathway. Drug Des, Dev Ther. (2017) 11(0):2787–99. 10.2147/DDDT.S140644 PMC5628699 29033541

[17] Feng T Yao J Chen Y Wang J Zhu Y Lan Z. Dexmedetomidine attenuates postoperative delirium by activating Nrf2 to reduce oxidative stress and blood-brain barrier disruption. Brain Res Bull. (2025) 230:111523. 10.1016/j.brainresbull.2025.111523 40858194

[18] Puls R von Haefen C Bührer C Endesfelder S. Dexmedetomidine protects cerebellar neurons against hyperoxia-induced oxidative stress and apoptosis in the juvenile rat. Int J Mol Sci. (2023) 24(9):7804. 10.3390/ijms24097804 37175511 PMC10178601

[19] He H Sun M Chen Y Zhou Y Qie W Tu W. Dexmedetomidine alleviates the hypoxic-ischemic brain damage via miR-20a-5p/methionine adenosyltransferase 2B axis in rat pups. NeuroReport. (2022) 33(5):205–14. 10.1097/WNR.0000000000001750 35287147

[20] Endesfelder S Makki H von Haefen C Spies CD Bührer C Sifringer M. Neuroprotective effects of dexmedetomidine against hyperoxia-induced injury in the developing rat brain. PLoS One. (2017) 12(2):e0171498. 10.1371/journal.pone.0171498 28158247 PMC5291450

[21] Ji D Karlik J. Neurotoxic impact of individual anesthetic agents on the developing brain. Children. (2022) 9(11):1779. 10.3390/children9111779 36421228 PMC9689007

[22] Engelhard K Werner C Eberspächer E Bachl M Blobner M Hildt E. The effect of the *α*2-agonist dexmedetomidine and the N-methyl-d-aspartate antagonist S(+)-ketamine on the expression of apoptosis-regulating proteins after incomplete cerebral ischemia and reperfusion in rats. Anesthesia Analg. (2003) 96(2):524–31. 10.1213/00000539-200302000-00041 12538207

[23] Pandharipande PP Pun BT Herr DL Maze M Girard TD Miller RR. Effect of sedation with dexmedetomidine vs lorazepam on acute brain dysfunction in mechanically ventilated patients: the MENDS randomized controlled trial. JAMA. (2007) 298(22):2644–53. 10.1001/jama.298.22.2644 18073360

[24] Walsh TS Parker RA Aitken LM McKenzie CA Emerson L Boyd J. Dexmedetomidine- or clonidine-based sedation compared with propofol in critically ill patients. JAMA. (2025) 334(1):32–45. 10.1001/jama.2025.7200 40388916 PMC12090071

[25] Sperotto F Mondardini MC Dell’Oste C Vitale F Ferrario S Lapi M. Efficacy and safety of dexmedetomidine for prolonged sedation in the PICU: a prospective multicenter study (PROSDEX)*. Pediatr Crit Care Me. (2020) 21(7):625–36. 10.1097/PCC.0000000000002350 32224830

[26] Aydogan MS Korkmaz MF Ozgül U Erdogan MA Yucel A Karaman A. Pain, fentanyl consumption, and delirium in adolescents after scoliosis surgery: dexmedetomidine vs midazolam. Pediatr Anesthesia. (2013) 23(5):446–52. 10.1111/pan.12128 23448434

[27] Garisto C Ricci Z Tofani L Benegni S Pezzella C Cogo P. Use of low-dose dexmedetomidine in combination with opioids and midazolam in pediatric cardiac surgical patients: randomized controlled trial. Minerva Anestesiol. (2018) 84(9):1053–62. 10.23736/S0375-9393.18.12213-9 29516704

[28] Mondardini MC Daverio M Caramelli F Conti G Zaggia C Lazzarini R. Dexmedetomidine for prevention of opioid/benzodiazepine withdrawal syndrome in pediatric intensive care unit: interim analysis of a randomized controlled trial. Pharmacother: J Hum Pharmacol Drug Ther. (2022) 42(2):145–53. 10.1002/phar.2654 34882826

[29] Gulla KM Sankar J Jat KR Kabra SK Lodha R. Dexmedetomidine vs midazolam for sedation in mechanically ventilated children: a randomized controlled trial. Indian Pediatr. (2021) 58(2):117–22. 10.1007/s13312-021-2124-7 33632940

[30] Zorko DJ Klowak JA Vu M Mayer YMZ. Pysklywec A Lewis K. Efficacy and safety of analgosedation with dexmedetomidine in critically ill mechanically ventilated children: a systematic review and meta-analysis of randomized controlled trials. Intensiv Care Med Paediatr Neonatal. (2025) 3(1):30. 10.1007/s44253-025-00091-4 PMC12460513 41019379

[31] Baserga M DuPont TL Ostrander B Minton S Sheffield M Balch AH. Dexmedetomidine use in infants undergoing cooling due to neonatal encephalopathy (DICE trial): a randomized controlled trial: background, aims and study protocol. Front Pain Res. (2021) 2:770511. 10.3389/fpain.2021.770511 PMC8915736 35295519

[32] Mencía S Palacios A García M Llorente AM Ordóñez O Toledo B. An exploratory study of sevoflurane as an alternative for difficult sedation in critically ill children. Pediatr Crit Care Me. (2018) 19(7):e335–41. 10.1097/PCC.0000000000001538 29557840

[33] Murphy KL Baxter MG. Long-Term effects of neonatal single or multiple isoflurane exposures on spatial memory in rats. Front Neurol. (2013) 4:87. 10.3389/fneur.2013.00087 23847588 PMC3703565

[34] Ing C Vutskits L. Unanswered questions of anesthesia neurotoxicity in the developing brain. Curr Opin Anaesthesiol. (2023) 36(5):510–5. 10.1097/ACO.0000000000001295 37552011 PMC10939468

[35] Broad KD Hassell J Fleiss B Kawano G Ezzati M Rocha-Ferreira E. Isoflurane exposure induces cell death, microglial activation and modifies the expression of genes supporting neurodevelopment and cognitive function in the male newborn piglet brain. PLoS One. (2016) 11(11):e0166784. 10.1371/journal.pone.0166784 27898690 PMC5127656

[36] McCann ME Soriano SG. Does general anesthesia affect neurodevelopment in infants and children? Br Med J. (2019) 367:l6459. 10.1136/bmj.l6459 31818811

[37] McCann ME de Graaff JC Dorris L Disma N Withington D Bell G. Neurodevelopmental outcome at 5 years of age after general anaesthesia or awake-regional anaesthesia in infancy (GAS): an international, multicentre, randomised, controlled equivalence trial. Lancet. (2019) 393(10172):664–77. 10.1016/S0140-6736(18)32485-1 30782342 PMC6500739

[38] Davidson AJ Disma N de Graaff JC Withington DE Dorris L Bell G. Neurodevelopmental outcome at 2 years of age after general anaesthesia and awake-regional anaesthesia in infancy (GAS): an international multicentre, randomised controlled trial. Lancet. (2016) 387(10015):239–50. 10.1016/S0140-6736(15)00608-X 26507180 PMC5023520

[39] Sun LS Li G Miller TLK Salorio C Byrne MW Bellinger DC. Association between a single general anesthesia exposure before age 36 months and neurocognitive outcomes in later childhood. Jama. (2016) 315(21):2312–20. 10.1001/jama.2016.6967 27272582 PMC5316422

[40] Warner DO Zaccariello MJ Katusic SK Schroeder DR Hanson AC Schulte PJ. Neuropsychological and behavioral outcomes after exposure of young children to procedures requiring general anesthesia. Anesthesiology. (2018) 129(1):89–105. 10.1097/ALN.0000000000002232 29672337 PMC6008202

[41] Ji SH Kang P Cho SA Park JB Jang YE Kim EH. Effects of dexmedetomidine–remifentanil on neurodevelopment of children after inhalation anesthesia: a randomized clinical trial. Anesthesiology. (2025) 143(4):827–34. 10.1097/ALN.0000000000005634 40923823

[42] Saynhalath R Disma N Taverner FJ von Ungern-Sternberg BS Andropoulos D Ng AS. Short-term outcomes in infants after general anesthesia with low-dose sevoflurane/dexmedetomidine/remifentanil versus standard-dose sevoflurane (the TREX trial). Anesthesiology. (2024) 141(6):1075–85. 10.1097/ALN.0000000000005232 39283983

[43] Reighard C Junaid S Jackson WM Arif A Waddington H Whitehouse AJO. Anesthetic exposure during childhood and neurodevelopmental outcomes. Jama Netw Open. (2022) 5(6):e2217427. 10.1001/jamanetworkopen.2022.17427 35708687 PMC9204549

[44] Russell AE Ford T Williams R Russell G. The association between socioeconomic disadvantage and attention deficit/hyperactivity disorder (ADHD): a systematic review. Child Psychiatry Hum Dev. (2016) 47(3):440–58. 10.1007/s10578-015-0578-3 26266467

[45] Durkin MS Yeargin-Allsopp M. Socioeconomic Status and pediatric neurologic disorders: current evidence. Semin Pediatr Neurol. (2018) 27:16–25. 10.1016/j.spen.2018.03.003 30293586 PMC8340602

[46] Gaynor JW Stopp C Wypij D Andropoulos DB Atallah J Atz AM. Neurodevelopmental outcomes after cardiac surgery in infancy. Pediatrics. (2015) 135(5):816–25. 10.1542/peds.2014-3825 25917996 PMC4533222

[47] Andropoulos DB Ahmad HB Haq T Brady K Stayer SA Meador MR. The association between brain injury, perioperative anesthetic exposure, and 12-month neurodevelopmental outcomes after neonatal cardiac surgery: a retrospective cohort study. Pediatr Anesthesia. (2014) 24(3):266–74. 10.1111/pan.12350 PMC4152825 24467569

[48] Ariyama J Hayashida M Shibata K Sugimoto Y Imanishi H O-oi Y. Risk factors for the development of reversible psychomotor dysfunction following prolonged isoflurane inhalation in the general intensive care unit. J Clin Anesthesia. (2009) 21(8):567–73. 10.1016/j.jclinane.2009.01.011 20122588

[49] Arnold JH Truog RD Rice SA. Prolonged administration of isoflurane to pediatric patients during mechanical ventilation. Anesthesia Analg. (1993) 76(3):520. 10.1213/00000539-199303000-00011 8452259

[50] Jabaudon M Quenot J-P Badie J Audard J Jaber S Rieu B. Inhaled sedation in acute respiratory distress syndrome. JAMA. (2025) 333(18):1608-17. 10.1001/jama.2025.3169 40098564 PMC11920880

[51] Miatello J Palacios-Cuesta A Radell P Oberthuer A Playfor S Amores-Hernández I. Inhaled isoflurane for sedation of mechanically ventilated children in intensive care (IsoCOMFORT): a multicentre, randomised, active-control, assessor-masked, non-inferiority phase 3 trial. Lancet Respir Med. (2025) 13(10):897–910. 10.1016/S2213-2600(25)00203-6 40680761

[52] Bose S Kelly L Shahn Z Novack L Banner-Goodspeed V Subramaniam B. Sedative polypharmacy mediates the effect of mechanical ventilation on delirium in critically ill COVID-19 patients: a retrospective cohort study. Acta Anaesthesiol Scand. (2022) 66(9):1099–106. 10.1111/aas.14119 35900078 PMC9353360

[53] Dai D Feinstein JA Morrison W Zuppa AF Feudtner C. Epidemiology of polypharmacy and potential drug–drug interactions among pediatric patients in ICUs of U.S. Children’s Hospitals and Pediatr Crit Care Me. (2016) 17(5):e218–28. 10.1097/PCC.0000000000000684 PMC5243142 26959349

[54] Escobar C Rojas-Granados A Angeles-Castellanos M. Chapter 16 development of the circadian system and relevance of periodic signals for neonatal development. Handb Clin Neurol. (2021) 179(Chronobiol Int 9 1992):249–58. 10.1016/B978-0-12-819975-6.00015-7 34225966

[55] Bathory E Tomopoulos S. Sleep regulation, physiology and development, sleep duration and patterns, and sleep hygiene in infants, toddlers, and preschool-age children. Curr Probl Pediatr Adolesc Heal Care. (2017) 47(2):29–42. 10.1016/j.cppeds.2016.12.001 28117135

[56] Goel P Goel A. Exploring the evolution of sleep patterns from infancy to adolescence. Cureus. (2024) 16(7):e64759. 10.7759/cureus.64759 39156264 PMC11329291

[57] Barclay NL Gregory AM. The neurobiology of childhood. Curr Top Behav Neurosci. (2013) 16:337–65. 10.1007/978-3-662-45758-0_239 24170426

[58] Carskadon Dement WC. Monitoring and staging human sleep. In: Kryger MH Roth T Dement WC, editors. Principles and Practice of Sleep Medicine. St. Louis: Elsevier Saunders (2011). p. 16–26.

[59] Tarokh L Saletin JM Carskadon MA. Sleep in adolescence: physiology, cognition and mental health. Neurosci Biobehav Rev. (2016) 70:182–8. 10.1016/j.neubiorev.2016.08.008 27531236 PMC5074885

[60] Byeon AG Weiss SK Gilfoyle E McKinnon NK. Sleep fragmentation in critically ill children: a review of contributing factors in the pediatric intensive care unit and neurodevelopmental outcomes. Front Sleep. (2025) 4:1629408. 10.3389/frsle.2025.1629408 41425199 PMC12713949

[61] Soto PJL Jiménez-Pastor JM López-Coleto L Cruz MME. Enhancing sleep quality in pediatric intensive care: a chronobiological perspective. Dent Clin North Am. (2024) 68(3):467–74. 10.1016/j.cden.2024.03.003 38879280

[62] Hassinger AB Afzal S Rauth M Breuer RK. Pediatric intensive care unit related sleep and circadian dysregulation: a focused review. Semin Pediatr Neurol. (2023) 48:101077. 10.1016/j.spen.2023.101077 38065630

[63] Alegria L Brockmann P Repetto P Leonard D Cadiz R Paredes F. Improve sleep in critically ill patients: study protocol for a randomized controlled trial for a multi-component intervention of environment control in the ICU. PLoS One. (2023) 18(5):e0286180. 10.1371/journal.pone.0286180 37228142 PMC10212109

[64] Manning JC Pinto NP Rennick JE Colville G Curley MAQ. Conceptualizing post intensive care syndrome in children—the PICS-p framework. Pediatr Crit Care Med. (2018) 19(4):298–300. 10.1097/PCC.0000000000001476 29406379

[65] Rosenberg L Traube C. Sedation strategies in children with pediatric acute respiratory distress syndrome (PARDS). Ann Transl Med. (2019) 7(19):509. 10.21037/atm.2019.09.16 31728362 PMC6828786

[66] Grasso C Marchesini V Disma N. Applications and limitations of neuro-monitoring in paediatric anaesthesia and intravenous anaesthesia: a narrative review. J Clin Med. (2021) 10(12):2639. 10.3390/jcm10122639 34203942 PMC8232784

[67] Ramaswamy SM Kuizenga MH Weerink MAS Vereecke HEM Nagaraj SB Struys MMRF. Do all sedatives promote biological sleep electroencephalogram patterns? A machine learning framework to identify biological sleep promoting sedatives using electroencephalogram. PLoS One. (2024) 19(7):e0304413. 10.1371/journal.pone.0304413 38954679 PMC11218986

[68] Lobo C Sivajohan A Vas N Keshvadi A Raheel H Iansavitchene A. pEEG scoping review. Can J Anesthesia. (2026).

[69] Ferguson H Sanjuan A Perkins AJ Elias M Wang S Mekala SN. Relationship between ICU delirium and change in quality of life, mood, and cognition over 12 months in survivors of acute respiratory failure. CHEST Crit Care. (2026) 4(1):100242. 10.1016/j.chstcc.2026.100242 41938652 PMC13048346

[70] Lachman SE Klein MJ Hamilton A Fuchs C Gold JI Nelson LP. Delirium and cognitive dysfunction in and beyond the pediatric intensive care unit. Pediatr Neurol. (2025) 173:182–90. 10.1016/j.pediatrneurol.2025.09.002 41175822

[71] Dervan LA Killien EY Smith MB Watson RS. Health-Related quality of life following delirium in the PICU*. Pediatr Crit Care Med. (2022) 23(2):118–28. 10.1097/PCC.0000000000002813 34534165 PMC8816806

[72] van den Boogaard M Kox M Quinn KL van Achterberg T van der Hoeven JG Schoonhoven L. Biomarkers associated with delirium in critically ill patients and their relation with long-term subjective cognitive dysfunction; indications for different pathways governing delirium in inflamed and noninflamed patients. Crit Care. (2011) 15(6):R297. 10.1186/cc10598 22206727 PMC3388649

[73] Fan YY Luo RY Wang MT Yuan CY Sun YY Jing JY. Mechanisms underlying delirium in patients with critical illness. Front Aging Neurosci. (2024) 16:1446523. 10.3389/fnagi.2024.1446523 39391586 PMC11464339

[74] Nashit M Haque AU Mirza S Jurair H Habib I Shahid S. Prevalence and risk factors of pediatric delirium in critically ill children: a study using cornell assessment of pediatric delirium tool at a tertiary care hospital. Indian J Crit Care Med: Peer-Rev, Off Publ Indian Soc Crit Care Med. (2025) 29(9):760–4. 10.5005/jp-journals-10071-25035 PMC12532215 41113532

[75] Bone JN Shen Y Harden S Retallack J Carwana M Murthy S. Socioeconomic Status, rurality, and pediatric critical care admission. JAMA Netw Open. (2026) 9(3):e263594. 10.1001/jamanetworkopen.2026.3594 41885861 PMC13022738

[76] Williams CN Eriksson CO Kirby A Piantino JA Hall TA Luther M. Hospital mortality and functional outcomes in pediatric neurocritical care. Hosp Pediatr. (2019) 9(12):958–66. 10.1542/hpeds.2019-0173 31776167 PMC6877428

[77] Ibrahim H Athar S Harhara T Elhag SA MElnour S Sukkar HH. Post-infectious and post-acute sequelae of critically ill adults with COVID-19. PLoS One. (2021) 16(6):e0252763. 10.1371/journal.pone.0252763 34138871 PMC8211258

[78] Choong K Fraser D Al-Harbi S Borham A Cameron J Cameron S. Functional recovery in critically ill children, the “WeeCover” multicenter study. Pediatr Crit Care Med. (2018) 19(2):145–54. 10.1097/PCC.0000000000001421 29394221

